# Metachronous B‐Cell Acute Lymphoblastic Leukemia After Localized Neuroblastoma Treated Without Cytotoxic Therapy: A Case Report

**DOI:** 10.1155/crh/5543785

**Published:** 2026-05-25

**Authors:** Reihaneh Sedghi, Ahmad Nazar Zadeh, Mohammadreza Bordbar, Omid Reza Zekavat, Shirin Parand

**Affiliations:** ^1^ Pediatric Hematology Department, Shiraz Medical School, Shiraz University of Medical Sciences, Shiraz, Iran, sums.ac.ir; ^2^ Pediatric Department, Shiraz University of Medical Sciences, Shiraz, Iran, sums.ac.ir; ^3^ Hematology Research Center, Shiraz University of Medical Sciences, Shiraz, Iran, sums.ac.ir

**Keywords:** acute lymphoblastic leukemia, leukemia cutis, neuroblastoma, second primary neoplasms, therapy-related leukemia

## Abstract

A 7‐month‐old infant developed therapy‐independent acute lymphoblastic leukemia (ALL) 8 months after complete resection of an International Neuroblastoma Risk Group (INRG) Stage L1 adrenal neuroblastoma (N‐MYC nonamplified) treated with surgery alone. The patient presented with leukemia cutis and discordant bone marrow findings: the initial aspirate revealed > 90% lymphoblasts (CD45+/CD19+/CD10+/TdT+), whereas two subsequent marrow examinations showed normocellular hematopoiesis, suggesting spatially heterogeneous marrow involvement with predominant extramedullary disease. Skin biopsy confirmed leukemic infiltration (PAX5+/TdT+/CD34+/CD10−). Germline testing was not available. Standard‐risk chemotherapy achieved complete remission, and the patient is currently receiving maintenance therapy. This case highlights that second primary ALL can occur in the absence of cytotoxic therapy after neuroblastoma and underscores the need for long‐term surveillance and consideration of genetic evaluation in selected patients.

**Key Clinical Message (KCM)**
• Second primary acute lymphoblastic leukemia can occur after neuroblastoma even in the absence of chemotherapy or radiotherapy. Discordant bone marrow findings may reflect patchy marrow involvement, and extramedullary disease can be diagnostically decisive. Long‐term surveillance and consideration of genetic evaluation are important in pediatric cancer survivors with atypical presentations.

## 1. Introduction

Second malignant neoplasms, including leukemia, are rare but serious complications in pediatric cancer survivors and are most commonly associated with exposure to chemotherapy or radiotherapy, particularly in children treated for neuroblastoma. While chemotherapy is a well‐established risk factor for treatment‐related leukemias, increasing evidence suggests that inherited genetic susceptibility and other factors may also play a role in the development of secondary malignancies. Neuroblastoma exhibits substantial biological heterogeneity, and germline pathogenic variants are increasingly recognized as determinants of tumor initiation, progression, and long‐term survivorship risks. Here, we report an infant who developed a second primary ALL following surgery alone for International Neuroblastoma Risk Group (INRG) Stage L1 neuroblastoma [1–3].

## 2. Case History and Examination

A 7‐month‐old infant presented with fever and diarrhea. Initial imaging revealed a heterogeneous mass in the right adrenal gland measuring 31 × 24.4 mm, with calcifications and minimal vascularity, raising concern for neuroblastoma or Wilms tumor. A spiral computed tomography (CT) scan confirmed a soft tissue lesion measuring 33 × 23 × 35 mm in the right adrenal gland, consistent with neuroblastoma. Metaiodobenzylguanidine (MIBG) scintigraphy demonstrated uptake in the upper right abdominal region and a suspicious focus in the right humerus. Following multidisciplinary tumor board review, the humeral uptake was interpreted as a false‐positive finding. The disease was therefore classified as INRG Stage L1 (Table 1), and the patient underwent complete surgical resection of the adrenal mass without adjuvant therapy. Genetic testing revealed a normal copy number of the N‐MYC oncogene. Bone marrow aspiration and biopsy performed at that time demonstrated normocellular marrow without dysplastic features, and the patient remained asymptomatic during follow‐up.

**TABLE 1:** INRG staging system (INRGSS) table [4].

INRGSS stage	Description	Key imaging/clinical criteria
Stage L1	Localized tumor not involving vital structures.	Confined to one body compartment (neck, chest, abdomen, or pelvis). No image‐defined risk factors (IDRFs) are present.
Stage L2	Locoregional tumor with involvement of vital structures.	Presence of one or more IDRFs. Tumor may be ipsilaterally continuous between two body compartments.
Stage M	Disseminated metastatic disease.	Distant metastases (e.g., to distant lymph nodes, bone, bone marrow [beyond MS criteria], organs). Not contiguous with the primary tumor.
Stage MS	“Special” metastatic disease in infants.	Metastatic disease in children younger than 18 months. Metastases are confined to skin, liver, and/or bone marrow (with negative MIBG scan in cortical bone).

Eight months after surgery, the infant developed fever and rash without initial evidence of leukemic infiltration. Laboratory evaluation revealed leukopenia (white blood cell count 1600/μL; neutrophils 1.2%; and lymphocytes 91.9%), anemia (hemoglobin 10 g/dL and mean corpuscular volume 60.8 fL), and thrombocytosis (platelet count 691,000/μL). Liver transaminases were elevated (AST 71 U/L and ALT 112 U/L), while inflammatory markers were within normal limits (C‐reactive protein 4.1 mg/L and erythrocyte sedimentation rate 42 mm/hr). Renal function tests, urinalysis, and abdominal–pelvic ultrasonography were unremarkable.

## 3. Methods: Differential Diagnosis, Investigations, and Treatment

Day 0: Diagnostic bone marrow aspiration and biopsy revealed > 90% lymphoblasts, establishing a diagnosis of pre‐B‐cell acute lymphoblastic leukemia (ALL). Flow cytometric immunophenotyping demonstrated CD45+, CD19+, and CD10+ blasts, with absence of myeloid markers. Cytogenetic analysis showed a normal karyotype. No antileukemic therapy, corticosteroids, or cytotoxic agents were administered between subsequent bone marrow examinations.

Day 10: Upon transfer to a tertiary referral center with parental consent, laboratory evaluation showed mild leukopenia (white blood cell count 2900/μL) with lymphocyte predominance (86%), hemoglobin 11.7 g/dL with persistent microcytosis, platelet count 632,000/μL, erythrocyte sedimentation rate 14 mm/h, lactate dehydrogenase 305 U/L, and uric acid 6.8 mg/dL. A repeat bone marrow aspiration and biopsy was obtained at a tertiary referral center to reassess disease status prior to initiation of therapy. Unexpectedly, this specimen demonstrated normocellular bone marrow with preserved trilineage hematopoiesis and no morphologic or flow‐cytometric evidence of blasts or residual neuroblastoma despite the absence of any intervening corticosteroid or antileukemic therapy. Given the improving peripheral blood counts, absence of clinical symptoms, and negative marrow findings, antileukemic therapy was deferred.

Day 53: Postauricular lymphadenopathy and multiple cutaneous scalp nodules developed. Histopathological examination of a skin lesion revealed dense dermal infiltration by monomorphic blast cells. Immunohistochemistry showed positivity for PAX5, TdT, CD34, and a high Ki‐67 proliferative index, with absence of CD10 expression, consistent with cutaneous involvement by a CD10‐negative immunophenotypic subclone of pre‐B ALL. Following receipt of the skin biopsy results, a repeat bone marrow aspiration and biopsy was performed to reassess marrow involvement.

Day 60: A repeat bone marrow aspiration and biopsy again demonstrated normocellular marrow with trilineage hematopoiesis and no evidence of leukemic involvement. Cerebrospinal fluid analysis was negative for malignant cells.

Overall, the initial bone marrow findings (CD19+/CD10+/TdT+) contrasted with the immunophenotype of the cutaneous lesion (CD19+/TdT+/CD34+/PAX5+/CD10–), indicating clonal heterogeneity. The presence of extramedullary disease, together with the initial diagnostic marrow findings, confirmed active systemic B‐cell ALL. The discordant marrow results were attributed to either sampling artifact or spatially heterogeneous (“patchy”) marrow involvement, a phenomenon that may result in false‐negative biopsies. Following multidisciplinary consensus, induction chemotherapy was initiated according to a standard‐risk Children’s Oncology Group (COG) protocol. This case report was prepared in accordance with the CARE guidelines [5].

## 4. Outcome and Follow‐Up

The patient achieved complete remission after induction, with minimal residual disease (MRD) testing by flow cytometry confirming negativity. Consolidation therapy was administered per protocol, followed by maintenance chemotherapy. Hematopoietic stem cell transplantation (HSCT) was not pursued, as the patient remained MRD‐negative throughout therapy. At present, the patient remains alive in complete remission at 17 months of follow‐up, without evidence of relapse or significant late complications.

## 5. Discussion

Neuroblastoma is the most common extracranial solid tumor of childhood and exhibits considerable clinical heterogeneity. According to the INRG staging system, Stage L1 disease represents a localized tumor confined to a single body compartment without image‐defined risk factors, for which complete surgical resection is often curative and adjuvant therapy is not required [3].

This case highlights the rare occurrence of a second primary ALL following surgery alone for Stage L1 neuroblastoma, without any prior exposure to chemotherapy or radiotherapy. Therapy‐related myelodysplasia and acute myeloid leukemia are well‐recognized complications of cytotoxic treatment, particularly with alkylating agents and topoisomerase II inhibitors, and secondary ALL has also been described in this context [3]. However, the absence of any cytotoxic exposure in this patient raises the possibility of an underlying genetic predisposition although this remains unproven due to the lack of germline testing.

Emerging data suggest that inherited genetic susceptibility can contribute to the development of second malignancies. Pathogenic variants in genes such as TP53, ALK, and PHOX2B have been implicated in cancer predisposition syndromes and genomic instability. [6–8]. In this patient, the absence of N‐MYC amplification further supports the involvement of alternative biological pathways. Epidemiological studies such as those by Applebaum et al. and Kamihara et al. have shown that survivors of neuroblastoma are at increased risk of various second malignancies, including renal cell carcinoma, thyroid cancer, and leukemia, reinforcing the importance of long‐term surveillance in this population [1, 6, 9].

An additional complexity in this case was the marked discrepancy between the initial diagnostic bone marrow sample and subsequent biopsies obtained prior to any antileukemic therapy. While marrow involvement in childhood acute leukemia is typically diffuse, rare instances of asymmetric or focal infiltration have been reported. For example, Yan et al. described two post‐HSCT patients in whom one iliac crest showed > 50% blasts while the opposite side showed 0%, and Hangai et al. reported pediatric ALL relapse with multifocal bone and soft tissue involvement, implying nonuniform marrow infiltration. Additionally, MRI studies in children indicate that marrow replacement is generally diffuse, with focal/patchy involvement being uncommon. Such findings highlight the potential for false‐negative biopsies and warrant consideration of bilateral or image‐guided sampling in diagnostically challenging cases. Such spatially heterogeneous involvement may lead to false‐negative biopsies. In the present case, the diagnostic dilemma was resolved by the identification of extramedullary disease, with skin biopsy confirming leukemic cutis (PAX5+/TdT+/CD34+), providing definitive evidence of persistent systemic B‐cell ALL despite negative marrow findings [6, 10]. The lack of CD10 expression in the cutaneous infiltrate, in contrast to the initial marrow blasts, indicates immunophenotypic divergence and clonal heterogeneity, underscoring the dynamic nature of leukemic evolution and the importance of integrating clinical, morphologic, and immunophenotypic data.

Beyond the acute management of second primary leukemias, structured survivorship and transition pathways from pediatric to adult healthcare services are crucial for childhood, adolescent, and young adult cancer survivors. Children with complex clinical courses or suspected genetic susceptibility require coordinated, multidisciplinary follow‐up extending into adulthood. In Italy, Zucchetti et al. have proposed a national, survey‐based transition protocol designed to standardize survivorship care and identify gaps in the transition from pediatric to adult services. This framework emphasizes the continuity of care, risk‐adapted surveillance, and collaboration between pediatric and adult providers and is particularly relevant for patients such as the one described here. Although this patient is an infant, the principles of structured survivorship care and transition planning remain applicable across the pediatric and adolescent–young adult continuum [11].

This case has several limitations, most notably the lack of comprehensive germline genetic testing and the inability to define the molecular basis of the observed immunophenotypic heterogeneity. Nevertheless, it contributes to the growing body of evidence that second malignancies in pediatric cancer survivors may arise independently of treatment exposure and highlights important diagnostic, biological, and survivorship considerations.

## 6. Conclusion

This case demonstrates that a second primary ALL can occur after neuroblastoma even in localized disease treated without cytotoxic therapy, raising concern for possible underlying susceptibility. It highlights the importance of long‐term surveillance, awareness of diagnostic pitfalls such as patchy marrow involvement, and consideration of genetic evaluation where available. Future research should focus on defining genetic risk factors to inform targeted follow‐up strategies for pediatric cancer survivors.

## Author Contributions

All authors contributed to the clinical management of the patient, data collection, and manuscript preparation.

## Funding

This study received no external funding.

## Disclosure

All authors have read and approved the final version of the manuscript. The corresponding author, Prof. Omid Reza Zekavat, had full access to all of the data in this study and takes complete responsibility for the integrity of the data and the accuracy of the data analysis.

## Ethics Statement

Ethical approval was not required for this case report in accordance with local/national institutional policy. This report was prepared in compliance with the CARE guidelines.

## Consent

Written informed consent for the publication of this case report and any accompanying images was obtained from the patient’s legal guardian.

## Conflicts of Interest

The authors declare no conflicts of interest.

## Supporting Information

Additional supporting information can be found online in the Supporting Information section.

## Supporting information


**Supporting Information** CARE Checklist: The CARE checklist has been completed and is submitted as a supplementary file.

## Data Availability

The data that support the findings of this case report are available from the corresponding author upon reasonable request.
