# Supplementary material for: Metachronous B‐Cell Acute Lymphoblastic Leukemia After Localized Neuroblastoma Treated Without Cytotoxic Therapy: A Case Report
Source: Case Rep Hematol. 2026 May 25;2026:5543785. doi: 10.1155/crh/5543785 (PMC13201254; doi:10.1155/crh/5543785)
Supplement: Supplementary file 1 — Supporting Information CARE Checklist: The CARE checklist has been completed and is submitted as a supplementary file. [file CRH-2026-5543785-s001.docx]

The CARE reporting checklist

For checking that clinical case report articles can be understood and used by everyone

|  | Item Description | Location (or reason for not reporting) |
| --- | --- | --- |
| **Sections** |  |  |
| [1. Title](https:/resources.equator-network.org/reporting-guidelines/care/items/title.html?utm_source=care&utm_medium=checklist&utm_campaign=CARE_2013_v1_1) | The area of focus and “case report” should appear in the title. | *Metachronous B-cell Acute Lymphoblastic Leukemia After Localized Neuroblastoma Treated Without Cytotoxic Therapy: A Case Report* |
| [2. Keywords](https:/resources.equator-network.org/reporting-guidelines/care/items/keywords.html?utm_source=care&utm_medium=checklist&utm_campaign=CARE_2013_v1_1) | The key elements of this case in 2–5 words. |  |

|  |
| --- |

| [3. Abstract](https:/resources.equator-network.org/reporting-guidelines/care/items/abstract.html?utm_source=care&utm_medium=checklist&utm_campaign=CARE_2013_v1_1) | 3a – Introduction: What does this case add? | Abstract, sentences 1 & 2 (context), and final 2 sentences |
| --- | --- | --- |
|  | 3b – Case presentation: | Abstract, sentences 2-6. |
|  | - The main symptoms of the patient(s). | "presented with leukemia cutis" |
|  | - The main clinical findings. | "discordant bone marrow findings", "Skin biopsy confirmed leukemic infiltration" |
|  | - The main diagnoses and interventions. | "therapy-independent acute lymphoblastic leukemia (ALL)", "Standard-risk chemotherapy" |
|  | - The main outcomes. | "Achieved complete remission", "patient is currently receiving maintenance therapy" |
|  | 3c – Conclusion: What are the main “take-away lessons from this case? | Abstract, final sentence. |
| [4. Introduction](https:/resources.equator-network.org/reporting-guidelines/care/items/introduction.html?utm_source=care&utm_medium=checklist&utm_campaign=CARE_2013_v1_1) | Brief background summary of the case referencing the relevant medical literature. | **Introduction Section**, entire paragraph. |
| [5a. Patient information](https:/resources.equator-network.org/reporting-guidelines/care/items/patient-information.html?utm_source=care&utm_medium=checklist&utm_campaign=CARE_2013_v1_1) | 5a – Demographic information of the patient (age, gender, ethnicity, occupation). | “A 7-month-old infant" |
|  | 5b – Main symptoms of the patient (chief complaint). | "presented with fever and diarrhea" (initial); later "developed fever and rash” |
|  | 5c – Medical, family, and psychosocial history—including lifestyle and genetic information whenever possible, details about relevant comorbidities, and past interv… | History of INRG stage L1 neuroblastoma, status post complete resection. No family or genetic history available. |
| [6. Clinical findings](https:/resources.equator-network.org/reporting-guidelines/care/items/clinical-findings.html?utm_source=care&utm_medium=checklist&utm_campaign=CARE_2013_v1_1) | Describe the relevant physical examination (PE) findings. | **Case History and Examination Section**, paragraph 2 (rash, fever). **Methods Section**, Day 53 (post-auricular lymphadenopathy, cutaneous scalp nodules). |
| [7. Timeline](https:/resources.equator-network.org/reporting-guidelines/care/items/timeline.html?utm_source=care&utm_medium=checklist&utm_campaign=CARE_2013_v1_1) | Depict important date and times in this case (table or figure). | **Methods Section** is structured chronologically (Day 0, Day 10, Day 53, Day 60). This serves as the narrative timeline. |
| [8. Diagnostic assessment](https:/resources.equator-network.org/reporting-guidelines/care/items/diagnostic-assessment-and-diagnosis.html?utm_source=care&utm_medium=checklist&utm_campaign=CARE_2013_v1_1) | 8a – Diagnostic methods (e.g., physical examination, laboratory testing, imaging, questionnaires) | Bone marrow aspiration/biopsy, flow cytometry, cytogenetics, CT, MIBG, skin biopsy, immunohistochemistry. |
|  | 8b – Diagnostic challenges (e.g., financial, language, or cultural) | Discordant bone marrow findings ("spatially heterogeneous marrow involvement") |
|  | 8c – Diagnostic reasoning including other diagnoses considered | Initial concern for neuroblastoma/Wilms tumor; resolution of ALL diagnosis via extramedullary disease confirmation. |
|  | 8d – Prognostic characteristics (e.g., staging) where applicable. | **Case History:** "INRG stage L1," "N-MYC non-amplified." **Methods:** "normal karyotype." |
| [9. Therapeutic Intervention](https:/resources.equator-network.org/reporting-guidelines/care/items/therapeutic-interventions.html?utm_source=care&utm_medium=checklist&utm_campaign=CARE_2013_v1_1) | 9a – Types of intervention (e.g., pharmacologic, surgical, preventive, self-care) | Surgery for neuroblastoma; standard-risk ALL chemotherapy protocol |
|  | 9b – Administration (e.g., dosage, strength, duration) | "Induction chemotherapy... according to a standard-risk Children’s Oncology Group protocol," followed by consolidation and maintenance |
|  | 9c – Changes in intervention (with rationale). | - |
| [10. Follow up and outcomes](https:/resources.equator-network.org/reporting-guidelines/care/items/follow-up-and-outcomes.html?utm_source=care&utm_medium=checklist&utm_campaign=CARE_2013_v1_1) | 10a – Clinician and patient-assessed outcomes | "Achieved complete remission," "MRD negative," "remains alive in complete remission at 17 months." |
|  | 10b – Important follow-up test results (positive and negative) | "Minimal residual disease testing... confirming negativity." |
|  | 10c – Intervention adherence and tolerability (and how this was assessed) | Implied by successful completion of phases and ongoing maintenance. |
|  | 10d – Adverse and unanticipated events. | without evidence of relapse or significant late complications." |
| [11. Discussion](https:/resources.equator-network.org/reporting-guidelines/care/items/discussion.html?utm_source=care&utm_medium=checklist&utm_campaign=CARE_2013_v1_1) | 11a – Strengths and limitations of the management of this case | Final paragraph: "This case has several limitations, most notably the lack of comprehensive germline genetic testing..." |
|  | 11b – Relevant medical literature | Throughout, with citations [1-4, 7-9] on second malignancies, genetics, patchy marrow involvement, survivorship. |
|  | 11c – Rationale for conclusions (including assessment of cause and effect) | Paragraphs 2 & 3 discuss the rarity and implication of no cytotoxic therapy; paragraph 4 analyzes discordant marrow findings. |
|  | 11d – Main “take-away” lessons of this case report. | **Conclusion Section**, entire. |
| [12. Patient perspective](https:/resources.equator-network.org/reporting-guidelines/care/items/patient-perspective.html?utm_source=care&utm_medium=checklist&utm_campaign=CARE_2013_v1_1) | When appropriate patients should share their perspectives on the treatments they received. | **Not Applicable.** The patient is an infant. The guardian provided consent, but a personal perspective cannot be obtained. |
| [13. Informed consent](https:/resources.equator-network.org/reporting-guidelines/care/items/informed-consent.html?utm_source=care&utm_medium=checklist&utm_campaign=CARE_2013_v1_1) | Did the patient give informed consent? Please provide if requested. | **Consent Statement** in Declarations: "Written informed consent for publication was obtained from the patient’s legal guardian." |
